# Iatrogenic Cushing’s Syndrome: The Result of Cobicistat and Glucocorticoid Interaction in an HIV Patient After Bariatric Surgery

**DOI:** 10.7759/cureus.34367

**Published:** 2023-01-30

**Authors:** Vânia Benido Silva, Joana Cardoso, Maria Esteves Brandão, Isabel Mesquita, Maria Teresa Pereira

**Affiliations:** 1 Department of Endocrinology, Centro Hospitalar Universitário do Porto, Porto, PRT; 2 Department of Infectious Diseases, Centro Hospitalar Universitário do Porto, Porto, PRT; 3 Department of Pneumology, Centro Hospitalar Universitário do Porto, Porto, PRT; 4 Department of General Surgery, Centro Hospitalar Universitário do Porto, Porto, PRT

**Keywords:** cyp3a4, iatrogenic cushing’s syndrome, hiv, cushing syndrome, fluticasone, interaction, cobicistat

## Abstract

Cobicistat, used as a pharmacokinetic booster in therapeutic combination with human immunodeficiency virus (HIV) protease inhibitors and integrase inhibitors, is a strong inhibitor of cytochrome P450 3A4 (CYP3A4). Since most glucocorticoids are metabolized by the isoenzyme of the cytochrome P450 pathway, their plasma concentrations can be highly increased in the presence of cobicistat-boosted darunavir, with subsequent risk of iatrogenic Cushing’s syndrome (ICS) and secondary adrenal insufficiency. We report a case of a 45-year-old man with HIV-hepatitis C virus co-infection treated with raltegravir and darunavir/cobicistat since 2019. In May 2021, he underwent a sleeve gastrectomy due to morbid obesity (BMI: 50.9 kg/m2) with multiple comorbidities. Four months after surgery, he was diagnosed with asthma and was started on inhaled budesonide, which was later changed to fluticasone propionate. At the 12-month postoperative visit, the patient referred proximal muscle weakness and asthenia, and suboptimal weight loss (excess weight loss of 39%) and high blood pressure were documented. Moon facies, buffalo hump, and abdominal large vinous striae were evident on physical examination. Laboratory studies showed impaired glucose metabolism and hypokalemia. Cushing’s syndrome was suspected and further investigation confirmed its iatrogenic origin. The diagnosis of ICS and consequent secondary adrenal insufficiency due to an interaction between the darunavir/cobicistat combination and budesonide/fluticasone was established. Darunavir/cobicistat therapy was replaced by dolutegravir/doravirine dual therapy, inhaled corticoid was switched to beclomethasone, and glucocorticoid substitutive therapy was introduced.

This is a particular case of overt ICS due to cobicistat-inhaled corticosteroid interaction in a superobese patient, developed after he underwent bariatric surgery. The presence of morbid obesity, combined with the rarity of this pharmacological complication in individuals taking cobicistat, made the correct diagnosis even more challenging.

A meticulous review of pharmacologic habits and potential interactions is essential to avoid serious harm to patients.

## Introduction

Cobicistat is a relatively recent drug for the treatment of human immunodeficiency virus (HIV) infection, without any antiretroviral activity but with a pharmacokinetic booster role [1]. It is used at subtherapeutic doses in combination with HIV protease inhibitors and integrase inhibitors (elvitegravir), allowing significant increases in their plasmatic concentrations to therapeutic levels with lower doses and frequencies and with fewer side effects [1,2]. Similar to other antiretrovirals, cobicistat is a potent inhibitor of cytochrome P450 3A4 (CYP3A4) activity, and the occurrence of potential pharmacological interactions and their harmful effects is a real problem in these patients [1].

A wide spectrum of respiratory pathology (asthma, chronic obstructive pulmonary disease (COPD), and rhinitis) is seen in HIV patients with a greater frequency than in the general population [3,4]. Therefore, these individuals often require treatment with inhaled corticosteroids, namely, fluticasone and budesonide. These drugs are metabolized by CYP3A4 in the liver, undergoing a first-pass effect, and under normal circumstances, they do not reach high plasma concentrations capable of causing systemic side effects [5]. However, the coadministration of a potent CYP3A4 inhibitor, such as cobicistat, can decrease the hepatic metabolism of exogenous corticosteroids, significantly increasing their half-life and systemic concentration, which consequently can culminate in the development of iatrogenic Cushing’s syndrome (ICS) [6,7]. Persistence of supraphysiologic cortisol levels may ultimately promote suppression of adrenocorticotropic hormone (ACTH) and endogenous corticosteroid secretion, resulting in secondary adrenal insufficiency (SAI) [6,7].

Although the interaction between these two pharmacological groups is well established in the literature, the development of overt clinical manifestations of Cushing's syndrome (CS) and its comorbidities in patients treated with cobicistat is a rare event [7,8]. In the presence of typical signs and symptoms of CS, a detailed review of medical history and pharmacological treatment is essential to assure a correct diagnosis.

## Case presentation

A 45-year-old Caucasian man with HIV-hepatitis C virus (HCV) co-infection diagnosed in 1999 has been treated with raltegravir 1200 mg per day and darunavir/cobicistat 800/150 mg per day since 2019. In the last evaluation with this antiretroviral therapy, his CD4+ T lymphocyte count was 802/mm3, with HIV-1 RNA replication of 43 copies/mL. In addition to this diagnosis, he also had super obesity (maximum weight: 150.5 kg; height: 1.72 m; BMI: 50.9 kg/m2) with multiple comorbidities, including hypertriglyceridemia, hyperuricemia, hepatic steatosis, osteoarticular pathology, and non-stratified lung disease. For that reason, after a clinical and analytical evaluation has been carried out to exclude secondary endocrine disorders for excess weight, in May 2021, the patient underwent bariatric surgery (sleeve gastrectomy) and was referred to our endocrinology outpatient clinic. Four months after surgery, when a 26 kg weight loss was achieved, he was diagnosed with asthma and was started on inhaled budesonide 200 ug per day, which was later changed to fluticasone propionate 92 ug per day (10 months after surgery).

**Figure 1:**
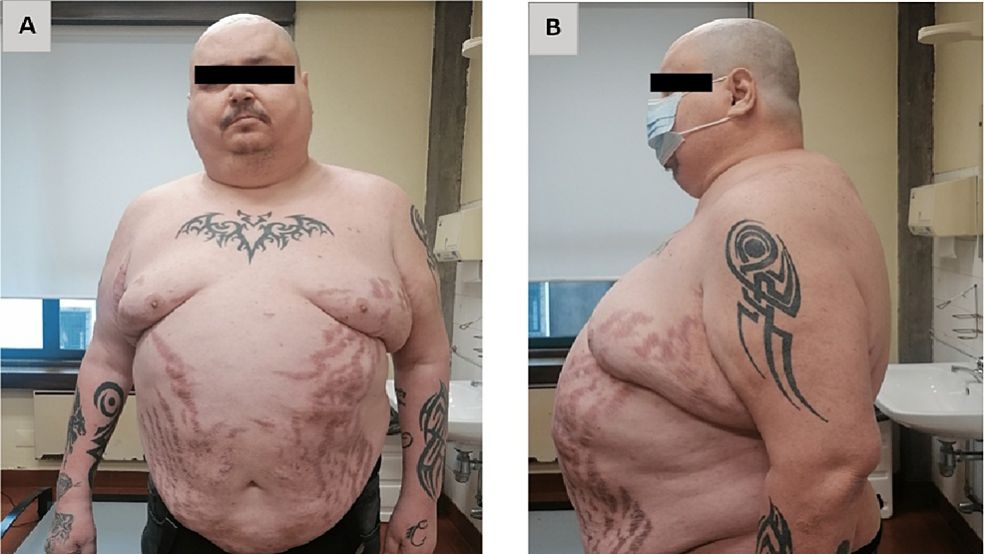
Hypercortisolism features in a 45-year-old man with iatrogenic Cushing's syndrome due to inhaled glucocorticoids and cobicistat interaction. (A) Moon facies and abdominal distention with large and lush vinous striae. (B) Dorsocervical fat accumulation (“buffalo hump”).

At the 12-month postoperative visit, the patient reported proximal muscle weakness with walking difficulties, progressive asthenia, and suboptimal weight loss (weight: 120.5 kg (-30 kg); excess weight loss of 39%) despite adherence to the nutritional plan. Also, a new diagnosis of hypertension had been documented. Physical examination revealed moon facies, a slight facial plethora, dorsocervical fat accumulation (“buffalo hump”), and abdominal distention with large and lush vinous striae (Figure 1). Laboratory studies showed impaired glucose metabolism (fasting glucose: 207 mg/dL; normal value: <100mg/dL) and hypokalemia of 3.03 mmol/L (normal range (NR): 3.5-5.0 mmol/L).

In view of these new recent clinical findings, CS was suspected, and further investigation confirmed its iatrogenic origin with a suppression of the hypothalamus-pituitary-adrenal (HPA) axis: undetectable 24-hours urinary free cortisol level (<9.6 ug/24 hours; NR: 4.3-176.0 ug/24 hours); suppressed morning and late-night salivary cortisol (both <0.054 ug/dL; NR: 0-0.783 ug/dL and 0-0.208 ug/dL, respectively); negative overnight 1 mg dexamethasone suppression test (morning cortisol 0.3 ug/dL; normal response: >5 ug/dL); low basal morning serum cortisol level (1.6 ug/dL; NR: 6.2-19.4 ug/dL); ACTH level of 7.81 pg/mL (NR: 9-52 pg/mL); and an inadequate response to adrenal stimulation test with 250 ug tetracosactide (serum cortisol levels of 4.0 ug/dL, 7.3 ug/dL, and 9.1 ug/dL at 0, 30, and 60 minutes, respectively; normal response: >18 ug/dL at any time). The diagnosis of ICS due to a pharmacologic interaction between the darunavir/cobicistat combination and budesonide/fluticasone was established.

Therefore, therapeutic adjustments were made: darunavir/cobicistat therapy was suspended and replaced by dolutegravir/doravirine dual therapy (which does not inhibit the CYP3A4 enzyme), inhaled corticoid was switched to beclomethasone, and glucocorticoid substitutive therapy with prednisolone 2.5 mg per day was introduced.

Two weeks later, the patient showed signs of some clinical improvement with less asthenia and muscle weakness, and objectively, a weight loss of 6 kg was observed.

## Discussion

ICS results from excessive and/or prolonged exposure to exogenous corticosteroids, mainly with the use of oral glucocorticoids and to a lesser extent with inhaled, topical, and injectable drugs [9]. However, multiple cases have contradicted the safety associated with inhaled corticosteroids by describing the development of CS due to their use, especially in HIV patients treated with CYP3A4 inhibitors [5,7-11].

Inhaled corticosteroids are the mainstay treatment for asthma. Budesonide and fluticasone are usually the first choices due to their favorable pharmacokinetic profile, including high lipophilicity, long half-life, and higher binding affinity for glucocorticoid receptors. Nonetheless, in the presence of a potent CYP3A4 inhibitor, these same properties facilitate the achievement of very high plasma concentrations [6,12]. Patients with HIV infection treated with the strongest CYP3A4 inhibitors, ritonavir or cobicistat, have a higher risk to develop this drug interaction [1,7].

The most frequently described pharmacological interaction with the greatest association with the development of CS is between fluticasone and ritonavir [5-7]. Our case, however, illustrates the rapid development of exuberant clinical manifestations compatible with ICS and consequent SAI about seven months after the introduction of budesonide and fluticasone in a patient with HIV infection treated with cobicistat. Although an increase in plasma cortisol levels is expected and observed by the co-administration of corticosteroids and cobicistat, the documentation of clinical cases with overt CS due to this interaction is rare [2,7,8,13].

Monge et al. reported a similar case of a 49-year-old man treated with darunavir/cobicistat who developed an ICS after two months of therapy with inhaled fluticasone due to COPD exacerbation [8]. A retrospective case-control study describing ICS due to corticosteroids and CYP3A4 inhibitors, recorded in the French Pharmacovigilance Database between 1996 and 2018, showed a much higher prevalence in HIV patients (97%) vs. the control group (7%). Of the 34 drug interactions in HIV patients, 31 occurred with ritonavir and only three with cobicistat. The main corticosteroid involved was inhaled fluticasone found in 80% [7].

Individuals with class III obesity have formal indications to undergo bariatric surgery, with improvement and/or resolution of their comorbidities [14]. After performing a gastric sleeve, a mean excess weight loss of 60% is expected [15]. When an inadequate response to the surgical procedure is documented, it is mandatory to exclude secondary causes, and it is extremely important to carry out an exhaustive anamnesis and careful physical examination. If this situation occurs in a patient with HIV infection under cobicistat and chronic airway disease, ICS due to drug interaction should always be considered [2]. In our case, at 12 months after surgery, the patient had only lost 39% of excess weight, and in the last assessment before the definitive diagnosis, he regained weight. After a complete review of his clinical history, it was confirmed that this unsatisfactory response to bariatric surgery coincided with the introduction of inhaled corticosteroids due to asthma and with the development of suggestive signs and symptoms of hypercortisolism. The establishment of clinical ICS in a superobese patient, short after gastric sleeve, can effectively compromise the potential benefit of weight loss with surgery.

In situations of ICS confirmed by this drug interaction, the most appropriate therapeutic management is not yet well established. Authors suggest switching the HIV treatment to a drug that does not inhibit CYP3A4 or changing the corticoid used for the respiratory disease, or both [2].

Ideally, patients treated with cobicistat should avoid the introduction of potent steroids metabolized via CYP3A4, including fluticasone, budesonide, and triamcinolone [2,7]. Beclomethasone is a safer alternative as it has a lower affinity for corticosteroid receptors and a shorter half-life, and it is predominantly metabolized by esterase hydrolysis, with CYP3A4 playing a minor role [2,16]. For this reason, adverse reactions due to systemic cortisol accumulation are not expected, even in the presence of potent CYP3A4 inhibitors [2,16]. If this selection is not possible, consideration should be given to switching cobicistat to another antiretroviral drug that does not interfere with CYP3A4, to prevent potential drug interactions, as was done in our patient [2].

Due to excess exogenous glucocorticoids, usually with treatments longer than three weeks, prolonged adrenal suppression and SAI can be established [2,17]. In these cases, after switching/discontinuing the drugs responsible for the interaction, complete recovery of the HPA axis may take from days to months [11]. For this reason, even before proceeding with the therapeutic adjustment, it is essential to start substitutive therapy with glucocorticoids to prevent the development of an adrenal crisis [2]. In our case, the patient started prednisolone 2.5 mg per day, and simultaneously, cobicistat was changed to dolutegravir/doravirine, and fluticasone was switched to beclomethasone. Even so, this management was sufficient for the attenuation of clinical manifestations and weight loss in just about two weeks, in a safe way and without any sign of hypocortisolism.

## Conclusions

To conclude, we present a particular case of overt ICS due to cobicistat-glucocorticoids interaction in a superobese patient, developed after he performed bariatric surgery, which may have compromised the results of the surgery. The presence of morbid obesity, a recognized cause of pseudo-Cushing, combined with the rarity of this pharmacological complication in individuals taking cobicistat makes diagnosis even more challenging.

This clinical case illustrates the importance of a meticulous review of all ongoing therapy before starting a new drug, especially when corticosteroid treatments are necessary for individuals on cobicistat. Anticipating potential complications and therapeutic interactions is essential for the best decisions and adjustments to be made to avoid serious harm to the patient.
